# Efficacy and safety of Androgen Deprivation Therapy (ADT) combined with modified docetaxel chemotherapy versus ADT combined with standard docetaxel chemotherapy in patients with metastatic castration-resistant prostate cancer: study protocol for a multicentre prospective randomized controlled trial

**DOI:** 10.1186/s12885-022-09276-y

**Published:** 2022-02-16

**Authors:** Xiangwei Yang, Hong Chen, Duanya Xu, Xianju Chen, Yamei Li, Jun Tian, Dongwen Wang, Jun Pang

**Affiliations:** 1 Department of Urology, Kidney and Urology Center, Pelvic Floor Disorders Center, The Seventh Affiliated Hospital, Sun Yat-sen University, Shenzhen, China; 2 School of Nursing, LKS Faculty of Medicine, University of Hong Kong, Hong Kong, China; 3 National Cancer Center/National Clinical Research Center for Cancer/Cancer Hospital & Shenzhen Hospital, Chinese Academy of Medical Sciences and Peking Union Medical College, Shenzhen, China

**Keywords:** Metastatic castration-resistant prostate cancer, Modified docetaxel chemotherapy, Androgen deprivation therapy

## Abstract

**Background:**

Androgen deprivation therapy (ADT) combined with docetaxel chemotherapy is the standard treatment for metastatic castration-resistant prostate cancer (mCRPC) patients. However, mCRPC patients are mainly frail elderly men, constantly accompanied by comorbidities and showing poor tolerance to standard docetaxel chemotherapy. Some exploratory studies administering modified chemotherapy regimens have reported noninferior oncologic outcomes with fewer adverse events, yet most are retrospective or small studies, and prospective randomized controlled trials have rarely been conducted. Therefore, we designed this modified docetaxel chemotherapy regimen in patients with mCRPC, aiming to evaluate its efficacy and safety compared with the standard docetaxel chemotherapy regimen.

**Methods:**

This is an open-label, multi-institutional, prospective, randomized non-inferiority trial. A total of 128 patients with mCRPC will be randomized to receive ADT combined with modified docetaxel chemotherapy (experimental group, *n*=64) or ADT combined with standard docetaxel chemotherapy (control group, *n*=64). Patients in the experimental group will receive a modified regimen with docetaxel 40 mg/m2 on the 1st day and 35 mg/m2 on the 8th day, repeated every 21 days. The primary endpoint is progression-free survival at 2 years. Secondary endpoints include overall survival, prostate-specific antigen response rate, pain response rate, toxicity and quality of life.

**Discussion:**

The expected benefit for the patient in the experimental arm is noninferior efficacy with decreased toxicity and improved quality of life compared with that in the control arm. To the best of our knowledge, this will be the first multicentre prospective randomized study to assess the efficacy and safety of modified docetaxel chemotherapy in patients with mCRPC in China. The results of this trial may provide benefit to mCRPC patients, especially those with poor performance.

**Trial registration:**

chictr.org.cn Identifier: ChiCTR2100046636 (May 24, 2021). Ongoing study.

## Background

Prostate cancer (PCa) was the second most commonly diagnosed cancer and the fifth leading cause of cancer death among men in 2020, with an estimated 1.4 million new cases and 375,000 deaths worldwide, accounting for 7.3% and 3.8% of all cancers diagnosed, respectively [1]. The incidence of PCa vary greatly across different regions, being highest in Northern and Western Europe, the Caribbean, Australia, New Zealand, Northern America and Southern Africa and lowest in Asia and Northern Africa [1].

In recent years, the incidence and mortality rates of PCa have been declining or stabilizing in many high-income countries while continuously rising in China [2–4]. The reasons for the incidence rise are unclear, but a more widespread use of prostate-specific antigen (PSA) testing, an increased use of transurethral resections and the fast advancement of ageing may play important roles [4–6]. The increase in mortality rates may be attributed to the high proportion of distant metastasis in newly diagnosed PCa patients. According to a multicentre study involving 525 PCa patients in China, as many as 54% had distant metastasis [7]. The 5-year relative survival rate is 30% in metastatic PCa patients compared with 80% in patients without distant metastases, and the progression-free survival (PFS) is half of that in patients who have not metastasized [8, 9]. The majority of patients with metastatic prostate cancer (mPC) are initially hormone-sensitive and gradually become castration-resistant as the disease progresses, and mCRPC is deemed to be the leading cause of death in PCa patients [10].

Androgen deprivation therapy (ADT) has been a cornerstone of treatment for metastatic PCa for decades [11], and its combination with docetaxel chemotherapy is recommended by the European Association of Urology (EAU) guidelines 2021 as the first-line standard treatment for mPC patients who can tolerate chemotherapy [12], based on the exciting results from TAX 327 and SWOG 9916 in patients with mCRPC and GET-AFU15, STAMPEDE, and CHAARTED in patients with metastatic hormone sensitive prostate cancer (mHSPC) [13–17].

Unfortunately, PCa patients, especially those with mCRPC, are mainly frail elderly men, and more than 40% of the newly diagnosed cases are over 75 years old [18], commonly accompanied by comorbid disorders such as hypertension, diabetes and coronary atherosclerotic heart disease. They show poor tolerance to standard docetaxel chemotherapy [19]. Therefore, various studies on modified regimens have been conducted, and favourable oncologic outcomes have been reported [20–23]. One of the most well-known studies conducted in mCRPC patients by Kellokumpu-Lehtinen Pirkko-Liisa et al. adopted a 2-week modified regimen by administering docetaxel 50 mg/m2 on the 1st and 15th days, repeated every 28 days. The efficacy of this 2-week regimen was not inferior to the standard 3-week regimen, and there were significantly fewer adverse events [20]. Chinese scholar Linjun Hu performed a retrospective study consisting of 50 mCRPC patients where 23 patients received modified regimen (docetaxel 40 mg/m2 on the 1st day and 35 mg/m2 on the 8th day, repeated every 21 days) [24], and 27 patients received standard regimen. The results showed that no significant difference was observed for overall survival (OS), PFS, pain response rate or PSA response rate between the two groups, while the incidence of grade 3 to 4 adverse events was significantly reduced in the modified regimen group.

A multicentre, prospective, randomized controlled trial concerning a modified docetaxel chemotherapy regimen in mCRPC patients has never been conducted in China. Hence, we designed this study, overcoming the shortcomings of previous studies in sample size, follow-up time and nonrandomized design, to explore the efficacy and safety of ADT combined with modified docetaxel chemotherapy versus ADT combined with standard docetaxel chemotherapy in patients with mCRPC. The result of this trial may benefit many mCRPC patients, especially those with poor performance status (PS) who cannot tolerate standard docetaxel chemotherapy.

## Methods/design

**Fig. 1:**
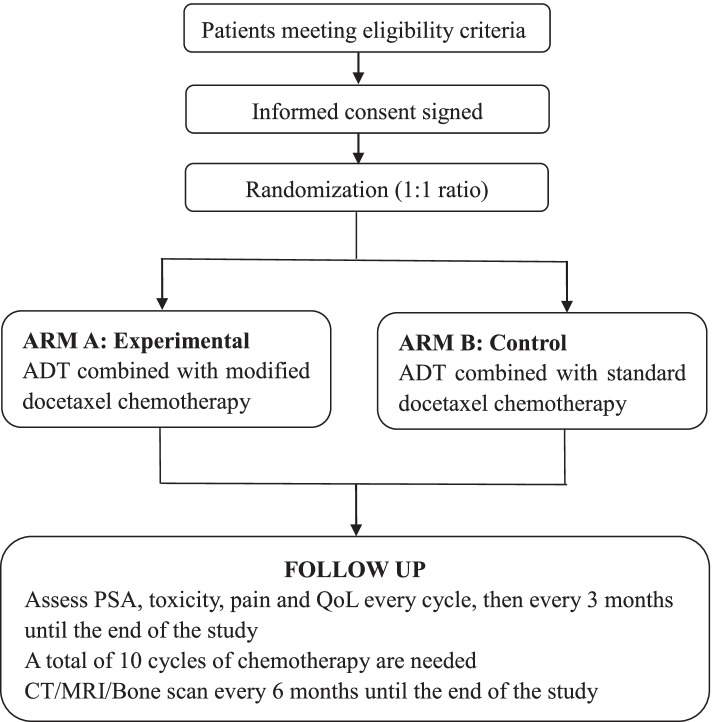
Flow chart of the study. ADT: Androgen deprivation therapy; PSA: Prostate-specific antigen; QoL: Quality of life; CT: Computed tomography; MRI: Magnetic resonance imaging

This is an open-label, multi-institutional, prospective, randomized non-inferiority study. Patients will be enrolled at two academic hospitals in China, including the Seventh Affiliated Hospital of Sun Yat-sen University and the National Cancer Center/National Clinical Research Center for Cancer/Cancer Hospital & Shenzhen Hospital, Chinese Academy of Medical Sciences and Peking Union Medical College. The recruiting clinicians will determine the eligibility of patients to participate in the trial. Considering the time and inconvenience of participating in the study, participants will be compensated ¥100 for the baseline enrollment visit and ¥100 for each return visit. Informed consent will be obtained from the patients, and then they will be randomized in a 1:1 ratio (Fig. 1):

- *Arm A (experimental group): ADT combined with modified docetaxel chemotherapy (docetaxel 40 mg/m2 on the 1st day and 35 mg/m2 on the 8th day, repeated every 21 days).*
- *Arm B (control group): ADT combined with standard docetaxel chemotherapy (docetaxel 75 mg/m2, repeated every 21 days).*

## Objectives

To assess the effect of ADT combined with modified docetaxel chemotherapy on PFS, OS, PSA response rate, pain response rate, toxicity and QoL in mCRPC patients compared with ADT combined with standard docetaxel chemotherapy.

### Primary endpoint

The primary endpoint is 2-year PFS. PFS is defined as the time from randomization until first evidence of disease progression or death from any cause [25]. The Prostate Cancer Working Group 3 (PCWG3) consensus will be used to define disease progression (more than 2 of 3 items of PSA progression, radiographic progression, and clinical progression). PSA progression is defined as a 25% increase over nadir with a minimum absolute level of 1 ng/ml [26], and radiographic progression will be evaluated according to the revised Response Evaluation Criteria in Solid Tumors (RECIST) guideline (version 1.1) [27]. Patients who are alive or lost to follow-up and those who have not experienced disease progression at the time of the evaluation will be censored [28].

### Secondary endpoint

The secondary endpoints are as follow:

- OS: OS is defined as the time from randomization to the date of documented death due to any cause [25]. Patients without documented death or lost to follow-up at the time of the final analysis will be censored [29].
- PSA response rate: The PSA response rate is defined as the percentage of patients with a PSA decline by ≥ 50% of the baseline level during treatment, which will be confirmed after ≥ 4 weeks by an additional PSA evaluation [30].
- Pain response rate: The pain response rate will be calculated as the proportion of responders among all participants. A Numeric Rating Scale (NRS) ranging from 0 to 10 will be used to assess pain [31]. A score of 0 means pain free, 1-3 means mild pain, 4-6 means moderate pain, 7-10 means severe pain. Responders include patients who experience a complete response or a partial response. A complete response is defined as an index pain score of 0 with no concomitant increase in the daily oral morphine equivalent dose (OMED) [32]. A partial response is defined as a reduction in pain score ≥ 2 without an increase in analgesic use or analgesic use reduction by ≥ 25% without an increase in pain score. Pain progression is defined as an increase of pain score ≥ 2 without a reduction in OMED or an increase by ≥ 25% in OMED without a decrease in pain score. An indeterminate response is defined as any response that does not qualify as a complete response, partial response, or pain progression.
- Toxicity will be assessed by the Common Terminology Criteria for Adverse Events (CTCAE) version 5.0 [33].
- QoL will be assessed by the Functional Assessment of Cancer Therapy-Prostate (FACT-P) [34, 35]. The FACT-P includes 22 general questions about physical, social, emotional, and functional wellbeing and 17 specific questions related to PCa. Each question is answered on a scale from 0 to 4 (0=not at all, 1=a little, 2=somewhat, 3=quite a lot, and 4=very much).

### Eligibility criteria

The inclusion criteria are as follows:

- Patients older than 18 years.
- Good general condition: Eastern Cooperative Oncology Group (ECOG) PS [36] ≤ 1.
- Patients with pathological proof of prostate adenocarcinoma, radiographic proof of distant metastasis, and resistance to castration therapy.
- Patients who are fully informed of the aims and procedures of the study and signed the informed consent form..

The exclusion criteria are as follows:

- Any medical conditions that preclude them from chemotherapy [20, 37] (allergic to docetaxel; blood neutrophil counts lower than 1.5 × 10^9^/L; platelet count lower than 100 × 10^9^/L; serum bilirubin concentration over 1.5 times the upper limit of normal; alanine or aspartate aminotransferase concentrations over 3 times the upper limit of normal; serum creatinine over 1.5 times the upper limit of normal).
- Previous history of chemotherapy with docetaxel.
- History of any cancers other than prostate cancer.
- Contraindication to CT/MRI/bone scan.
- Enrolled in another therapeutic trial.
- Any psychological, familial, sociological or geographical condition hampering compliance with the study protocol and follow-up schedule..

### Evaluation and randomization

Prior to randomization, a complete history inquiry, physical examination and histological confirmation of prostate adenocarcinoma are required, together with proof of distant metastasis and castration resistance. General information (age, body mass index (BMI), smoking and drinking history, medical and surgical history, comorbidities, blood-cell counts, blood biochemistry, PS status, pain score, QoL, etc.) and tumor-related information (digital rectal examination (DRE) results, PSA level, testosterone level, Gleason score [38], International Society of Urological Pathology (ISUP) grade [38], Tumor node metastasis (TNM) classification [39], EAU risk group [12], tumor volume assessed by CHAARTED criteria [17], etc.) will be recorded in detail. Standard operating procedures (SOPs) will be developed, providing step-by-step instructions on how to perform the study properly and detailing how data should be recorded on case report forms (CRFs). All source document and laboratory report data will be reviewed by two investigators to ensure accuracy and integrity.

The mechanism of implementing the allocation sequence (simple randomization) was generated by a clinical research assistant with Statistical Product and Service Solutions (SPSS) software (version 23.0). The study investigator will then receive an envelope with an allocation sequence to randomize each participant.

### Interventions/treatments

Participants in Arm A will receive ADT combined with a modified docetaxel chemotherapy regimen (docetaxel 40 mg/m2 on the 1st day and 35 mg/m2 on the 8th day, repeated every 21 days), and participants in Arm B will receive ADT combined with a standard docetaxel chemotherapy regimen (docetaxel 75 mg/m2, repeated every 21 days). Usually, ADT consists of bilateral orchiectomy and long-acting luteinizing hormone releasing hormone (LHRH) agonists or antagonists. Considering the low acceptance of orchiectomy and the absence of long-term depot formulations of LHRH antagonists, a 3-month depot formulation of LHRH agonists (triptorelin or goserelin) will be used to maintain a castration level. Dexamethasone (8 mg orally) will be administered 12 h, 3 h, and 1 h prior to instillation of docetaxel, and prednisone (10 mg daily) will be given during the period of chemotherapy. A total of 10 cycles of chemotherapy are recommended according to the current oncological national recommendations and international guidelines [37]. All participants will receive systemic standard of care for metastases, such as zoledronic acid, to reduce skeletal-related adverse events. Colony-stimulating factors (CSFs) will not be used unless grade 3 or 4 haematological toxic effects occur. In case of grade 4 haematological or grade 3 or higher nonhaematological toxic effects, the dose of docetaxel in the control group will be reduced to 60 mg/m2.

### Study duration and follow-up

The present research project will be sustained for 4 years, including 2 years for enrolment and 2 years for follow-up, during which specific milestones will be achieved and documented (Table 1). At each visit, the clinicians will conduct a history taking and physical examination and assess the PSA response rate, pain response rate, toxicities, and QoL. After discontinuation of the protocol treatments, the participants will be followed up every 3 months until disease progression or death or the end of the study. Chest and abdominal CT, pelvic MRI and bone scans will be performed every 6 months. Every patient will be followed up for at least 2 years if no progression or death is observed during this period. Additional examinations and treatments will be carried out at the discretion of the clinicians.

**Table 1:** Timeline and follow up of the study

	Screening	Treatment	Follow-up	
		**Every cycle**	**Every 3 months**	**Every 6 months**
**Patient consent**	×			
**History & physical**	×	×	×	
**Blood-cell counts**	×	×		
**Blood biochemistry**	×	×		
**PSA test**	×	×	×	
**Testosterone level**	×	×	×	
**CT/MRI**	×			×
**Bone scan**	×			×
**Pain assessment**	×	×	×	
**Toxicity assessment**	×	×	×	
**QoL**	×	×	×	

*PSA *Prostate-specific antigen, *CT *Computed tomography, *MRI *Magnetic resonance imaging, *QoL *Quality of life

### Measurement of response

PFS will be measured as the time from randomization to either progression or death, whichever occurs first. OS will be measured as the time from randomization to death due to any cause. Efficacy and safety evaluations will be performed at baseline, every cycle, and subsequently every 3 months until the end of the study.

### Statistical analysis

Sample size calculation: in a previous study, the median PFS was 6.3 months in the control arm [13]. We assumed 2.8 months as the noninferiority margin, 2 years for enrolment and 2 years for follow-up, and the randomization ratio was 1:1. Accepting a one-tailed type I error of 5% and 90% power (type II error of 10%), 103 patients need to be recruited. Considering a maximum dropout rate of approximately 20%, a total of 128 patients will need to be included.

Data analysis: SPSS 23.0 will be used for statistical analysis. Two research staff members will participate in the statistical process. Frequencies of events will be analysed using Pearson’s chi-square test or Fisher’s exact test, and continuous variables will be analysed by Student’s t-test or nonparametric tests in case of a nonnormal distribution. The median PFS and OS will be estimated with the Kaplan-Meier method, and survival between groups will be compared using the log-rank test. Patients without progression and alive at the time of analysis will be censored at the time of the latest assessment. A Cox proportional hazards regression model will be used to analyse the association between the survival time of the patients and the predictor variables, and the hazard ratio will be given with its 95% confidence interval. The tested differences will be considered significant if the p value is less than 0.05. Multiple imputations will be adopted to address any missing data.

### Data safety monitoring committee

A Data Safety Monitoring Committee (DSMC) [40] comprised of two clinicians knowledgeable in the field of the treatment of mCRPC, two statisticians and an ethicist will be set up to guarantee effective protection of the participants and to maintain the highest quality of the scientific data. The DSMC members will not be directly involved in the conduct of the trial directly. The DSMC will assess the safety and efficacy of the data, the study progress, protocol compliance, and data integrity for the study every 6 months. If safety concerns arise, more frequent meetings may be held. The DSMC has only a consultative role; it may recommend an early termination of the trial in cases of unacceptable toxicity, and the investigator will decide whether the DSMC recommendations will be followed.

## Discussion

Previous studies have shown the superiority of docetaxel over estramustine and mitoxantrone in patients with mPC [13–17]; consequently, ADT combined with docetaxel chemotherapy is recommended as the first-line standard treatment for mPC patients [12]. The second-generation taxane cabazitaxel did not demonstrate superiority for OS over docetaxel in chemotherapy-naïve mCRPC patients and thus is regarded as a second-line regimen [12, 41]. Metronomic chemotherapy, which is intended to prevent tumor angiogenesis based on more frequent and low-dose drug administrations, has achieved some clinical benefits in multiple clinical trials, with fewer adverse events observed [42, 43]. Nevertheless, a review involving the latest clinical trials of metronomic chemotherapy showed that the development of metronomic chemotherapy faces terra incognita; it seemed unlikely metronomic chemotherapy alone could achieve satisfactory results, and thus optimal combination regimens need to be explored [44]. Platinum-based chemotherapy regimens have shown some efficacy in patients with mCRPC, especially those with mutations in DNA damage repair (DDR) genes (BRCA2, BRCA1, ATM, etc.), whereas alterations in DDR genes occur in merely 25% of mCRPC patients [45]. Moreover, for patients with progression after abiraterone treatment, docetaxel is still capable of achieving modest PSA responses and PFS benefits [46], although cross-resistance between abiraterone and docetaxel may have a detrimental impact [47]. Docetaxel plays an important role in the treatment of mPC.

However, docetaxel is associated with various toxic effects, such as neutropenia, fatigue, nausea, vomiting, and diarrhoea [13]. Neutropenia is the most frequent haematologic toxicity and the most serious [48]. In the TAX 327 trial, grade 3-4 neutropenia was seen in 32% of patients [13], and the incidence could be even higher at 72% according to a Scandinavian trial [49]. In the 2-week regimen and the Chinese modified regimen mentioned above, the incidence of grade 3-4 neutropenia was significantly lower than that in the standard 3-week regimen, without compromising the chemotherapy efficacy [20, 24]. Elderly patients are generally more susceptible to haematologic toxicity, and they suffer grade 4 neutropenia more commonly than younger patients when treated with docetaxel at a dose of 75 mg/m2 [50]. In elderly patients with good performance status (ECOG-PS ≤ 1), docetaxel-based chemotherapy may be effective and feasible, while in patients with ECOG-PS higher than 1, its feasibility is unclear [51]. A case-control retrospective study involving 2514 patients suggested that chemotherapy offered no benefit in OS and possibly worsened the quality of life in patients with poor performance status (ECOG-PS ≥ 2) [52]. Our modified chemotherapy regimen may provide a new solution to these patients with poor performance status if the expected benefits are achieved in mCRPC patients with good performance status.

Tolerance to docetaxel may be distinct between different populations. According to a multicentre randomized clinical trial in China [53], the incidence of all adverse events and grade 3-4 neutropenia was 94.6% and 57.7%, respectively, in mCRPC patients treated with docetaxel, which is much higher than that in the TAX 327 study [13]. A 2-week regimen might work for Western population, but may not be the most suitable modified regimen for Chinese patients. We designed this multicentre, prospective, randomized controlled trial comparing the efficacy and safety of a modified docetaxel chemotherapy regimen with a standard docetaxel chemotherapy regimen in Chinese mCRPC patients to evaluate this issue. The results are awaited to compliment the abundance of existing studies and to benefit more mCRPC patients, potentially providing an innovative way to solve the intolerance problems that preclude many elderly mPC patients with poor performance status from receiving chemotherapy treatment.

## Data Availability

Data sharing is not applicable to this article as no datasets yet generated or analyzed during this ongoing study.

## Abbreviations

ADT: Androgen deprivation therapy
mCRPC: Metastatic castration-resistant prostate cancer
PCa: Prostate cancer
PSA: Prostate-specific antigen
PFS: Progression-free survival
mPC: Metastatic prostate cancer
EAU: European Association of Urology
mHSPC: Metastatic hormone sensitive prostate cancer
OS: Overall survival
PS: Performance status
LHRH: Luteinizing hormone releasing hormone
CSFs: Colony-stimulating factors
CT: Computed tomography
MRI: Magnetic resonance imaging
QoL: Quality of life
PCWG: Prostate Cancer Working Group
RECIST: Response Evaluation Criteria in Solid Tumors
NRS: Numeric Rating Scale
OMED: Oral morphine equivalent dose
CTCAE: Common Terminology Criteria for Adverse Events
FACT-P: Functional Assessment of Cancer Therapy-Prostate
ECOG: Eastern Cooperative Oncology Group
BMI: Body mass index
DRE: Digital rectal examination
ISUP: International Society of Urological Pathology
TNM: Tumor node metastasis
SOPs: Standard operating procedures
CRFs: Case report forms
SPSS: Statistical Product and Service Solutions
DSMC: Data Safety Monitoring Committee
DDR: DNA damage repair
GCP: Good Clinical Practice

## Declarations

Potential risks from the intervention: ADT combined with docetaxel 75 mg/m2 is the standard of usual care for patients with mCRPC, and adverse events are inherent to standard-of-care treatments, including neutropenia, fatigue, nausea, vomiting, diarrhoea, etc. Patients administered a modified regimen may suffer fewer adverse events at the cost of chemotherapy efficacy, which means that more effective treatments will be delayed and they may in consequence have a worse prognosis. A risk control plan was designed to prevent and cope with potential unexpected adverse events due to the study interventions. The study has to be stopped in case severe adverse events and disease progression occur due to the implementation of the intervention.
